# Ferritin Levels in Colombian Children: Findings from the 2010 National Nutrition Survey (ENSIN)

**DOI:** 10.3390/ijerph13040405

**Published:** 2016-04-05

**Authors:** Robinson Ramírez-Vélez, Jorge Enrique Correa-Bautista, Javier Martínez-Torres, Katherine González-Ruíz, Felipe Lobelo

**Affiliations:** 1 Centro de Estudios para la Medición de la Actividad Física «CEMA», Escuela de Medicina y Ciencias de la Salud, Universidad del Rosario, Bogotá D.C. 11001000, Colombia; jorge.correa@urosario.edu.co (J.E.C.-B.); epid_javier@unipamplona.edu.co (J.M.-T.); 2 Programa de Fisioterapia, Facultad de Salud, Universidad Manuela Beltrán, Bogotá D.C. 111321, Colombia; kt_gonxa89@hotmail.com; 3 Hubert Department of Global Health, Rollins School of Public Health, Emory University, Atlanta, GA 30306, USA; rlobelo@emory.edu

**Keywords:** nutrition, children, ferritin, prevalence

## Abstract

Low ferritin is associated with many adverse health outcomes and is highly prevalent worldwide. The aim of this study was to describe the key findings related to plasma ferritin levels to identify the prevalence and associated sociodemographic factors in a representative sample of children in Colombia, based on the 2010 National Nutrition Survey. We analyzed cross-sectional data from 6650 Colombian children between the ages of 5 and 12. Plasma ferritin levels were determined by chemiluminescence. Sociodemographic data was assessed by computer-assisted personal interview technology. All analyses were conducted considering the complex nature of the sample. Of the children assessed, 3.5% had low ferritin, defined as levels <12 µg/L. A multivariate logistic regression analysis revealed increased risks for low ferritin levels among black or Afro-Colombian ethnic group and for those living in the northern, western and southern regions of the country. In conclusion, a significant prevalence of anemia caused by low ferritin levels was found and various sociodemographic factors were associated with this finding in Colombia. Continued surveillance and implementation of interventions to improve dietary patterns among the identified high-risk groups should be considered. Implementing these recommendations can help reduce manifestations of iron deficiency (e.g., delays in infant and child development) and thus improve public health.

## 1. Introduction

Micronutrient deficiencies are more prevalent in developing countries and are typically due to inadequate food intake, poor dietary quality, and/or low bioavailability of nutrients, among other causes [1,2]. To estimate the magnitude of this problem, the use of direct indicators to assess various nutrient levels, such as vitamin A, zinc and vitamin B12, and iron has been proposed [3,4]. Iron stores in the body exist primarily in the form of ferritin and can be assessed through several laboratory tests. Hematological tests based on characteristics of red blood cells (*i.e.*, Hb concentration, hematocrit, mean cell volume, and red blood cell distribution width) are generally more available and less expensive than are biochemical tests (*i.e.*, erythrocyte protoporphyrin concentration, serum ferritin concentration, and transferrin saturation), tipically used to detect ealier changes in iron status [5].

The prevalence of anemia as a predictor of iron deficiency has also declined, thus decreasing the effectiveness of routine anemia screening among children. Currently, there are few global reports on the prevalence of iron deficiency in children, in particular for low-to-middle income countries experiencing rapid nutrition transitions such as Latin America [6]. Among preschool children (aged 1–5 years), iron-deficiency anemia results in developmental delays and behavioral disturbances (e.g., decreased motor activity, social interaction, and attention to tasks. Anaemia is a condition in which the number of red blood cells (and consequently their oxygen-carrying capacity) is insufficient to meet the body’s physiologic needs [5]. In turn, iron deficiency is defined as as a condition in which there are no mobilizing iron stores, causing a compromised supply of iron to tissues, including the red cells [1,5,6,7]. According to the World Health Organization (WHO) and Centers for Disease Control and Prevention (CDC) [7,8], iron deficiency constitutes 40%–50% of anemia cases (iron-deficiency anemia defined as a Hb concentration of less than or equal to 10.0 g/dL) in older children and up to 80% in preschool children (2–5 years). Other causes of anemia include parasites, chronic sub-clinical bleeding, chronic kidney disease, celiac disease and poorly absorbed (non-heme iron) [5,6,7]. Mujica-Coopman *et al.* [9] reported in a systematic review that highest prevalence of anemia (ranging from 7.6% to 18.7%) in children under 6 years of age was found in countries of Latin America and the Caribbean (on the basis of one of three abnormal values for erythrocyte protoporphyrin concentration, serum ferritin concentration and/or transferrin saturation). In Colombia, according to estimates from the National Survey of Nutritional Status [10], 2 in 10 children under 5 years of age are at risk for iron deficiency. In general, the prevalence of iron deficiency is higher among children living at or below the poverty level than among those living above the poverty level and higher among black or Mexican-American children than among white children [11,12].

The multiplicity of physiologic processes involving ferritin and in particular, its role as an acute phase reactant, has caused some to question the use of serum ferritin as a marker for the risk of various disorders [4,5,6,7,8]. This reinforces the hypothesis of iron as a potentially causal factor, with serum ferritin acting as a reliable marker of available reactive iron [8,10]. In addition, iron-deficiency anemia is associated with conditions that may independently affect infant and child development (*i.e.*, low birthweight, generalized undernutrition, poverty, and high blood level of lead) that need to be taken into account when interventions addressing iron-deficiency anemia are developed and evaluated [11,12]. Other consequences include compromised neurological development of children, increased maternal and infant mortality, and a reduction in physical work capacity in adulthood [13]. These effects may be irreversible; therefore, primary prevention through detection of iron deficiency in non-anemic individuals is paramount and justifies the need for this study. However, to the best of our knowledge, associations of iron deficiency with various socio-demographic factors that could help identify risk groups and offer information to better design interventions has not been investigated in a nationally representative sample in the Americas [14].

Therefore, this study was to describe the key findings related to plasma ferritin levels to identify the prevalence of low ferritin and associated sociodemographic factors in a representative sample of children in Colombia, based on the 2010 National Nutrition Survey.

## 2. Materials and Methods

### 2.1. Study Design and Participants

The Colombian National Nutrition Survey (*in Spanish* ENSIN, Encuesta Nacional de la Situación Nutricional en Colombia) was conducted in 2010 by the Colombian Institute of Family Welfare [10,15]. Details of the survey have been published elsewhere [10,15]. In brief, participants were selected to represent 99% of the country’s population using a multistage stratified sampling scheme. All municipalities from the thirty-two departments in the country were grouped into strata based on similar geographic and socio-demographic characteristics. One municipality was randomly chosen from each stratum, with probability proportional to the population size. Clusters of about ten households each were then randomly chosen from within these strata and household members were invited to participate. The survey included 50,670 households, representing 4987 clusters from 258 strata. Of the 7266 children aged to 5–12 years, a subsample 6650 (91.5%) were considered for the analysis. The first author applied to the PROFAMILIA-ENSIN and obtained permission to use the publicly available data for research and teaching learning purposes. Further details can be obtained from the website of PROFAMILIA-ENSIN [10].

### 2.2. Ethical Considerations

The study was conducted according to the guidelines laid down in the Declaration of Helsinki. All participants provided written informed consent and the Research Ethics Review Board at the Colombian Institute of Family Welfare approved the survey protocol. A comprehensive verbal description of the nature and purpose of the study and its experimental risks was given to all participants and, for participants under 18, their parents/guardians. All participants and parents/legal guardians of participants under 18 provided written informed consent before entering the study. The Ethical Committee of the PROFAMILIA provided ethical approval prior to data collection (Convenio No. 096 de 2009 ICBF). To conduct the present analysis using the ENSIN 2010 database, the Manuela Beltrán University Institutional Review Board exempted the project (Resolución 8430 de 1993; Ministerio de Salud de Colombia).

### 2.3. Blood Sample Collection, Serum Extraction, and Laboratory Analysis

In a subsample of participants, blood samples were obtained by venipuncture from the antecubital vein, and centrifuged within 0.5–1 h of collection. Serum was extracted and aliquoted immediately into screw-top vials. The samples were transported in iceboxes, protected from direct light, and stored in liquid nitrogen until processing at the National Institute of Health of Colombia.

Serum ferritin was determined by chemiluminescence, using an automatic analyzer (ADVIA Centaur^®^, Bayer Diagnostics, Tarrytown, NY, formerly Chiron Diagnostics, East Walpole, MA, USA). Low serum ferritin (SF) level was used as an indicator of iron deficiency and the cut-off for depleted iron stores was defined as SF < 12 µg/L. Our analysis excludes children with levels of C-reactive protein above 1.2 mg/d [15]. All participants in this study had non-fasting blood samples. However there may be some variability in ferritin levels depending on the time the sample was drawn in reference to the time after the last meal.

### 2.4. Sociodemographic Characteristics

The following socio-demographic variables were included as potentially associated factors with low ferritin levels: age (5 to 5.9, 6 to 6.9, 7 to 7.9, 8 to 8.9, 9 to 9.9, 10 to 10.9, 11 to 11.9 and 12 to 12.9); sex (male and female); urban category [grouped as urban and rural); ethnicity grouped as: (a) Indigenous; (b) Black or Afro-Colombian and (c) Others (mestizo); geographic region: (a) Atlantic (North); (b) Eastern; (c) Central; (d) Pacific (West); (e) Bogota and (f) national territories (south); and social or socio-economic status determined by the System of Identifying Potential Beneficiaries of Social Programs (SISBEN for its Spanish initials) (1 to 3, and 4 or more). The SISBEN is a system designed by the Colombian National Government to identify families who could benefit from social programs. It takes into account sociodemographic characteristics (family composition, employment status, family income, and educational level), living conditions (construction type and materials), and access to public utilities (sewer, electricity, potable water, and garbage collection). Households are classified into six levels with 1 being the poorest and 6 being the wealthiest. For this study we classified SISBEN scores into four categories (1–4 or more).

### 2.5. Statistical Analysis

First, we conducted an exploratory analysis of the frequency distribution (measures of central tendency and dispersion for quantitative variables) and relative frequencies (for qualitative variables) using the Pearson χ^2^ test with and without Yates correction. To estimate the relationship between low ferritin levels and sociodemographic variables in children (age, sex, urban category, geographic region, ethnicity and socioeconomic level-SISBEN), binary logistic regression models were used. The first adjusted model was for age and sex, the second model was based on ethnic group, geographic area, socioeconomic levels and urban category; the third model was adjusted by age, sex, ethnic group, geographic area, socioeconomic levels and urban category. Odds ratios were considered a confounder if they shifted the model in a constant direction with a proportional increase in the exposure level of at least 10%. All analyses were conducted with the use of the complex survey design routines of the SPSS Statistical software package version 20 (SPSS, Inc., Chicago, IL, USA).

## 3. Results

The study cohort consisted of 6650 children between 5 and 12 years of age (mean age 8.6 years). The range of ferritin levels was 40.1 (95% CI 39.2 to 41.0). A total of 3.5% of infants had ferritin levels below 12 µg/L. Children were 7 years old, that live in the Atlantic (North) area, and from specific ethnic groups (Afro-Colombian) had the highest percentage of ferritin levels values less than 12 µg/L (5.0%, 5.2% and 6.0%, respectively). The distribution of ferritin levels in children aged 5 to 12 years is shown in Table 1 and Table 2.

Table 3 shows the results for a logistic regression analysis. Being 7 years of age OR 2.52 (95% CI 1.50 to 4.22), 9 years of age OR 1.86 (95% CI 1.06 to 3.27) *or* 11 years of age OR 2.39 (95% CI 1.45 to 3.93), being part of the Black *or* Afro-Colombian ethnic groups OR 1.81 (95% CI 1.22 to 2.68), living in the pacific (west) OR 2.16 (95% CI 1.25 to 3.75) *or* Atlantic region (north) OR 2.52 (95% CI 1.52 to 4.16) and low socioeconomic status (level I SISBEN) OR 1.82 (95% CI 1.10 to 3.04) were predisposing factors for low ferritin levels (adjusted model by age, sex, ethnic group, geographic area, socioeconomic levels and urban category).

## 4. Discussion

According to the WHO 1993–2005 estimation of the worldwide prevalence of anemia, one-fourth of the total population (24.8%; 95% CI 22.9% to 26.7%) had this condition; the prevalence reaches 47.4% (95% CI 45.7% to 49.1%) in preschoolers and 30.2% (95% CI 28.7% to 31.6%) in non-pregnant women [15]. Mujica-Coopman *et al.* [9] reported among children 19 to 72 months of age, prevalence of anemia (4%) in Chile and Costa Rica (anemia defined as a Hb concentration of less than or equal to 10.0 g/dL *or* ferritin levels below 12 µg/L, respectively). In this study, we found that 3.5% of children in Colombia had a low ferritin levels. Because this result is above 3%, low ferritin levels can be characterized as a country-wide public health problem in this subgroup [9]. This prevalence is similar than previously reported for other countries, including Brazil [16], Nigeria [17] and Thailand [18]. In addition, the prevalence of low ferritin levels in our study was similar to results from a Euro-Growth study in European children that found 7.2% of 12 year-old children were iron deficient [19]. In accordance to previous reports from Colombia [10] and other countries [20], the highest prevalence of low ferritin levels was found in children from 7 to 12 years at 5% (95% CI 3.2 to 6.5) and 4.7% (95% CI 3.3 to 5.9) respectively, with no major differences by sex.

Serum ferritin, which represents only a small fraction of total ferritin, appears to be a marker for levels of active iron [21]. Serum ferritin concentration is an early indicator of the status of iron stores and is the most specific indicator available of depleted iron stores, especially when used in conjunction with other tests to assess iron status [22]. Under normal conditions, a direct relationship exists between serum ferritin concentration and the amount of iron stored in the body [23], such that 1 µg/L of serum ferritin concentration is equivalent to approximately 10 mg of stored iron [24]. However, measured ferritin levels have some limitation. Serum ferritin is a widely available enzyme-linked assay, which can be performed on a nonfasting blood sample. It is important to remember that elevated ferritin does not equal iron overload and there are many patients with elevated ferritin caused by inflammatory disease and subclinical infection. Acute and chronic inflammation can increase serum ferritin levels and the assessment of iron status but is not a cause iron deficiency [25].

On the other hand, our results show an association between low ferritin levels and multiple sociodemographic factors, including being between 5 and 12 years of age, living in the coastal region (Atlantic or Pacific) and belonging to an Afro-Colombian ethnic group, similar results published by investigators from the nutrition department of the WHO [7,8]. These associations have also been reported in Malaysia [26] and Kazakhstan [27]. Both of these studies showed that low ferritin levels were associated with poverty, overcrowding and a lack of public services, thus making children more vulnerable [28]. Although not measured in this study, in the Pacific and Atlantic regions, school meals are likely to have low phytate levels, because they consist mainly of fast food, eggs and milk [29]. In bean-consuming communities, the high iron level in the biofortified varieties coupled with the relatively high bean consumption and modest iron absorption indicate that beans should be a good vehicle for iron biofortification, despite their high phytic acid content [30]. Although iron absorption from beans is relatively low, biofortified beans have a much higher iron level than regular beans. A daily consumption of 100 g biofortified beans provide about 10 mg iron, a 5 mg increase in daily iron intake as compared to consuming the same quantity of regular beans [31]. In addition, in Colombia, the Pacific and Atlantic regions have higher levels aridity, high food prices, limited natural resources, and poor infrastructure development, which often affect the availability of and access to food and health services [32]. Such factors can lead to increased malnutrition among populations residing at this region.

Our study had some limitations. Our survey was about the health status of Colombian children and only ferritin levels was measured. The gold standard for determination of depleted iron stores is the lack of stainable iron in the bone marrow [33]. However, this is an invasive and costly examination. Less invasive laboratory tests such as determination of serum iron, transferrin, transferrin saturation, ferritin, soluble transferrin receptor, soluble transferrin receptor index and reticulocyte parameters are available and are proposed as useful in detection of iron depletion before the onset of anaemia [34]. However, except for ferritin [35] these tests are considered too expensive and time consuming and have not all been validated against stainable iron in the bone marrow [36]. A second limitation of the present study was that dietary intake of iron was not assessed. In the dietary study we conducted in Villamor [30], only 13.4% of the protein in the diets of children was derived from eggs and milk, with 40% of protein coming from meat. Colombia is a country that is geographically, climatically and ethnically diverse [10,15]. Clearly these differences could affect food supply, dietary practices, and consequently, iron intakes [37]. The Pacific, Eastern and Central parts of Colombia are very diverse, which lead to different dietary patterns and climate [38]. Lastly, we did not determine parasitic load. Several studies shown the regulation of iron distribution serves as an innate immune mechanism against invading pathogens. It is well known that the relationship between malnutrition and infection is an intimate one, and it is often associated with impaired immune function.

## 5. Conclusions

In conclusion, there is a significant prevalence of anemia due to low ferritin levels in Colombian children and various sociodemographic factors are associated with this condition. This problem could be addressed by tailored supplementation in clinical settings or via broader public health supplementation strategies as done in other countries such as Guatemala, Puerto Rico and México [1,9,12]. For example, the latest Colombian national survey indicated that only 32% of children over 6 months of age regularly consumed “Bienestarina”, a complementary food fortified with iron (14.1 mg/100 g) and multiple vitamins [39]. In addition, coordination with other dietary interventions and educational strategies can produce viable and sustainable interventions in particular among the high-risk groups identified in this study. The Colombian Institute of Family Welfare (ICBF, for its initials in Spanish) can help prevent and control iron deficiency by counseling individuals and families about sound iron nutrition during infancy, by screening persons on the basis of their risk for iron deficiency, and by treating and following up persons with presumptive iron deficiency. This practice would support the Millenium Development Goals [4] to promote the health of mothers and infants in developing countries [40].

## Figures and Tables

**Table 1:** Ferritin levels acording sociodemographic factors in Colombian children (National Nutrition Survey, 2010).

Characteristics	Ferritin Levels, µg/L	
	*n*	Mean (95% CI)
**Total**	6650	40.1 (39.2–41.0)
**Age (years)**		
5–5.9	743	36.0 (33.9–38.0)
6–6.9	735	36.9 (34.7–39.0)
7–7.9	809	39.0 (36.8–41.2)
8–8.9	843	42.4 (38.5–46.3)
9–9.9	850	42.4 (39.6–45.3)
10–10.9	930	41.3 (39.1–43.4)
11–11.9	905	41.3 (39.1–43.5)
12–12.9	835	40.3 (37.9–42.7)
**Sex**		
Male	3391	40.2 (38.8–41.6)
Female	3259	40.0 (38.3–41.2)
**Socioeconomic levels by SISBEN**		
Level I	4010	40.8 (39.4–42.2)
Level II	829	38.6 (36.1–41.0)
Level III	566	39.4 (37.1–41.6)
Level IV	1245	39.8 (38.1–41.4)
**Geographic area**		
Atlantic (North)	1474	38.7 (37.2–40.2)
Eastern	952	42.3 (40.6–44.0)
Central	1454	43.1 (40.4–45.7)
Pacific (Western)	965	37.2 (35.5–38.9)
Bogotá	291	38.3 (36.1–40.6)
National territories (South)	1514	38.0 (34.2–41.8)
**Urbanicity**		
Urban	4001	39.8 (38.8–40.9)
Rural	2649	40.7 (39.0–42.5)
Ethnic group *		
Indigenous	875	37.2 (34.5–39.9)
Black *or* Afro-Colombian	743	39.9 (37.2–42.5)
Others	4979	40.3 (39.3–41.3)

SISBEN, System of Identifying Potential Beneficiaries of Social Programs. * All children analysed by ethnic group were *n* = 6597, another 53 appertained to “Raizal del archipielago” and “Palenquero de San Basilio”, who were not analysed because this group do not have a representative sample.

**Table 2:** Characterization of ferritin levels among Colombian children (National Nutrition Survey, 2010).

Characteristics	Ferritin Level Deficiency <12 µg/L		Adequate Ferritin Levels >12 µg/L	
	*n*	% ** (95% CI)	*n*	% ** (95% CI)
**Total**	257	3.5 (3.0–3.9)	6393	96.5 (96.4–96.6)
**Age (years) ^a^**				
5–5.9	35	3.5 (2.3–4.6)	708	96.5 (96.2–96.7)
6–6.9	27	3.4 (1.9–4.6)	708	96.6 (96.4–96.8)
7–7.9	39	5.0 (3.2–6.5)	770	95.0 (94.7–95.2)
8–8.9	31	1.9 (1.1–2.5)	812	98.1 (98.0–98.3)
9–9.9	19	2.3 (1.1–3.3)	831	97.7 (97.6–97.8)
10–10.9	33	4.0 (2.4–5.4)	897	96.0 (95.8–96.2)
11–11.9	26	4.0 (2.4–5.4)	879	96.0 (95.8–96.2)
12–12.9	47	4.7 (3.3–5.9)	788	95.3 (95.0–95.6)
**Sex**				
Male	162	3.2 (2.6–3.8)	4001	96.8 (96.6–96.9)
Female	95	3.7 (3.0–4.3)	2649	96.3 (96.2–96.4)
**Socioeconomic levels by SISBEN**				
Level I	170	4.0 (3.3–4.7	3840	96.0 (95.8–96.1)
Level II ^a^	29	2.8 (1.8–3.7)	800	97.2 (97.0–97.4)
Level III ^a^	15	2.0 (0.9–2.8)	551	98.0 (97.9–98.2)
Level IV	43	3.2 (2.2–4.2)	1202	96.8 (96.6–96.9)
**Geographic area**				
Atlantic (North)	75	5.2 (4.1–6.2)	1399	94.8 (94.6–95.0)
Eastern ^a^	19	1.7 (0.8–2.4)	933	98.3 (98.2–98.4)
Central ^a^	55	2.4 (1.8–3.0)	1399	97.6 (97.4–97.7)
Pacific (Western) ^a^	44	5.0 (3.7–6.2)	921	95.0 (94.8–95.1)
Bogotá ^a^	9	3.1 (1.2–4.6)	282	96.9 (96.7–97.1)
National territories (South) ^a^	55	3.7 (2.5–4.8)	1459	96.3 (96.1–96.4)
**Urbanicity**				
Urban	162	3.4 (2.8–4.0)	3839	96.6 (96.5–96.7)
Rural	95	3.5 (2.7–4.2)	2554	96.5 (96.3–96.6)
Ethnic group *				
Indigenous ^a^	37	4.7 (2.3–6.7)	838	95.3 (95.0–95.5)
Black *or* Afro-Colombian	41	6.0 (4.3–7.6)	702	94.0 (93.7–94.2)
Others	177	3.0 (2.5–3.5)	4802	97.0 (96.9–97.1)

SISBEN, System of Identifying Potential Beneficiaries of Social Programs. * All children analysed by ethnic group were *n* = 6597, another 53 appertained to “Raizal del archipielago” and “Palenquero de San Basilio”, who were not analysed because this group do not have a representative sample. ** It is not correct to calculate the percentages from the “*n*” presented in this table; these calculations were taken from weighted values given to each participant. ^a^ Coefficient of variation is >20% deficiency prevalence, therefore variation should be used with caution.

**Table 3:** Sociodemographic factors associated with ferritin deficiency in Colombian children (National Nutrition Survey, 2010).

Characteristics	Bivariate	Adjusted Model ^a^	Adjusted Model ^b^	Adjusted Model ^c^
**Age (years) ^d^**				
5–5.9	**1.92 (1.01–3.67)**	**1.92 (1.01–3.67)**	**1.64 (0.96–2.81)**	**1.64 (0.96–2.81)**
6–6.9	1.82 (0.91–3.65)	1.83 (0.91–3.67)	1.48 (0.81–2.68)	1.48 (0.82–2.69)
7–7.9	**2.77 (1.46–5.28)**	**2.78 (1.46–5.29)**	**2.51 (1.50–4.22)**	**2.52 (1.50–4.22)**
9–9.9	1.23 (0.58–2.64)	1.24 (0.58–2.65)	1.11 (0.59–2.07)	1.11 (0.60–2.08)
10–10.9	**2.19 (1.12–4.26)**	**2.19 (1.12–4.26)**	**1.86 (1.06–3.27)**	**1.86 (1.06–3.27)**
11–11.9	1.55 (0.78–3.07)	1.56 (0.79–3.09)	1.38 (0.78–2.44)	1.39 (0.78–2.45)
12–12.9	**2.58 (1.40–4.77)**	**2.60 (1.41–4.80)**	**2.38 (1.44–3.91)**	**2.39 (1.45–3.93)**
**Sex ^e^**				
Female	**1.14 (0.82–1.59)**	**1.14 (0.82–1.59)**	**1.12 (0.83–1.51)**	**1.12 (0.84–1.50)**
**Socioeconomic levels by SISBEN ^f^**				
Level I	**2.10 (1.10–3.99)**	**2.12 (1.11–4.05)**	**1.81 (1.07–3.07)**	**1.82 (1.10–3.04)**
Level II	1.44 (0.68–3.03)	1.45 (0.68–3.06)	1.37 (0.73–2.55)	1.37 (0.75–2.51)
Level IV +	1.67 (0.82–3.42)	1.71 (0.84–3.51)	1.66 (0.92–3.00)	1.70 (0.96–3.01)
**Geographic area ^g^**				
Atlantic (North)	**3.19 (1.73–5.88)**	**3.16 (1.71–5.83)**	**2.57 (1.52–4.34)**	**2.52 (1.52–4.16)**
Central	1.44 (0.76–2.72)	1.42 (0.75–2.68)	1.20 (0.70–2.06)	1.18 (0.71–1.98)
Pacific (West)	**3.08 (1.62–5.82)**	**3.05 (1.62–5.76)**	**2.21 (1.24–3.94)**	**2.16 (1.25–3.75)**
Bogotá	1.86 (0.78–4.40)	1.82 (0.78–4.29)	1.55 (0.70–3.44)	1.50 (0.70–3.21)
National territories (South)	**2.24 (1.14–4.41)**	**2.23 (1.13–4.38)**	**1.70 (0.92–3.15)**	**1.68 (0.93–3.02)**
**Urbanicity ^h^**				
Rural	1.03 (0.73–1.44)	1.02 (0.73–1.44)	0.99 (0.74–1.33)	0.98 (0.74–1.31)
**Ethnic group ^i^**				
Indigenous	1.61 (0.87–2.97)	1.76 (0.99–3.12)	1.18 (0.66–2.11)	1.24 (0.70–2.20)
Black *or* Afro-Colombian	**2.08 (1.37–3.15)**	**2.49 (1.74–3.56)**	**1.79 (1.19–2.70)**	**1.81 (1.22–2.68)**

SISBEN, System of Identifying Potential Beneficiaries of Social Programs. Odds ratios (95% confidence interval), significant odds ratios are shown in bold. **^a^** adjusted by age and sex. **^b^** adjusted by ethnic group, geographic area, socioeconomic levels and urbanicity. **^c^** adjusted by age, sex, ethnic group, geographic area, socioeconomic levels and urbanicity. **^d^** reference group: 8–8.9. **^e^** reference group: Male. **^f^** reference group: Level III. **^g^** reference group: Eastern. **^h^** reference group: Urban. **^i^** reference group: Others.
